# Distal Dendritic Enrichment of HCN1 Channels in Hippocampal CA1 Is Promoted by Estrogen, but Does Not Require Reelin

**DOI:** 10.1523/ENEURO.0258-18.2018

**Published:** 2018-10-15

**Authors:** Maurice Meseke, Florian Neumüller, Bianka Brunne, Xiaoyu Li, Max Anstötz, Theresa Pohlkamp, Meike M. Rogalla, Joachim Herz, Gabriele M. Rune, Roland A. Bender

**Affiliations:** 1 Institute of Neuroanatomy, University Medical Center, Hamburg 20246, Germany; 2 Institute of Structural Neurobiology, Center of Molecular Neurobiology, Hamburg 20246, Germany; 3 Department of Molecular Genetics, University of Texas Southwestern Medical Center, Dallas, TX 75390

**Keywords:** Aromatase, estrogen, GPER1, hippocampus, neurosteroid, Reelin

## Abstract

HCN1 compartmentalization in CA1 pyramidal cells, essential for hippocampal information processing, is believed to be controlled by the extracellular matrix protein Reelin. Expression of Reelin, in turn, is stimulated by 17β-estradiol (E2). In this study, we therefore tested whether E2 regulates the compartmentalization of HCN1 in CA1 via Reelin. In organotypic entorhino-hippocampal cultures, we found that E2 promotes HCN1 distal dendritic enrichment via the G protein–coupled estrogen receptor GPER1, but apparently independent of Reelin, because GST-RAP, known to reduce Reelin signaling, did not prevent E2-induced HCN1 enrichment in distal CA1. We therefore re-examined the role of Reelin for the regulation of HCN1 compartmentalization and could not detect effects of reduced Reelin signaling on HCN1 distribution in CA1, either in the (developmental) slice culture model or in tamoxifen-inducible conditional *reelin* knockout mice during adulthood. We conclude that for HCN1 channel compartmentalization in CA1 pyramidal cells, Reelin is not as essential as previously proposed, and E2 effects on HCN1 distribution in CA1 are mediated by mechanisms that do not involve Reelin. Because HCN1 localization was not altered at different phases of the estrous cycle, gonadally derived estradiol is unlikely to regulate HCN1 channel compartmentalization, while the pattern of immunoreactivity of aromatase, the final enzyme of estradiol synthesis, argues for a role of local hippocampal E2 synthesis.

## Significance Statement

Mechanisms of learning and memory require a fine-tuned interplay between ion channels on hippocampal neurons. Among these, CA1 pyramidal cells have a crucial position, as they integrate information from various hippocampal and extrahippocampal sources. Identifying factors that influence the function of ion channels, such as the HCN1 channels, which filter entorhinal synaptic input, may therefore help to better understand processes that underlie learning and memory. Here we show that the dendritic localization of HCN1 channels, a critical determinant of their function, is influenced by estrogen (E2), thus bringing attention to the modulatory roles of sex hormones on hippocampal information processing, which are yet poorly understood.

## Introduction

Hyperpolarization-activated cyclic nucleotide-gated (HCN) channels generate the h-current (I_h_), a critical determinant of the resting membrane potential in neurons (Pape, 1996; Robinson and Siegelbaum, 2003). A variety of additional functions of I_h_ in neurons exist, but these depend strongly on the subcellular localization of the channels (Bender and Baram, 2008; Shah, 2014). For instance, in cortical pyramidal cells, HCN channels are strikingly enriched in the distal dendrites, but almost absent in the proximal dendritic and somatic compartments (Lörincz et al., 2002; Notomi and Shigemoto, 2004; Brewster et al., 2007). In hippocampal CA1, this distal dendritic enrichment of HCN channels in the pyramidal neurons filters synaptic input from the perforant path (Vaidya and Johnston, 2013) and supports the temporal integration of excitation in the CA1 pyramidal cells (Magee, 1999; Poolos et al., 2002). As HCN channels thus markedly influence hippocampus-dependent learning and memory (Nolan et al., 2004), an understanding of the mechanisms that govern their subcellular localization is of substantial interest, and contributing factors have previously been identified (Piskorowski et al., 2011; Lewis et al., 2011; Wilkars et al., 2012); however, without yet solving the question comprehensively.

In this context, a recent study proposed that the extracellular matrix protein Reelin promotes the distal dendritic enrichment of channels of the HCN1 subtype, the predominant cortical HCN isoform, in pyramidal cells (Kupferman et al., 2014). Reelin is best known for its role in brain development, during which it controls neuronal migration and cortical lamination (D’Arcangelo et al., 1995; Ogawa et al., 1995; Tissir and Goffinet, 2003; Förster et al., 2006). However, as some neurons continue to express Reelin in adult brain, its functions go beyond developmental roles and include the regulation of synaptogenesis and long-term potentiation (Herz and Chen, 2006; Bock and May, 2016; Lee and d’Arcangelo, 2016). The canonical Reelin signaling cascade involves the binding of a Reelin dimer to low-density lipoprotein receptors, the APOE receptor 2 (APOER2), and the very-low-density lipoprotein (VLDL) receptor, resulting in phosphorylation of the adaptor protein Disabled 1 (Dab1), which activates a variety of downstream signaling molecules (Bock and May, 2016; Lee and d’Arcangelo, 2016). Kupferman et al. (2014) showed that a viral knockdown of Dab1 results in a reduction of HCN1 channels and I_h_ in the distal dendritic compartment of CA1 and neocortical pyramidal cells *in vivo*. Similar results were observed in organotypic hippocampal cultures, if receptor-associated protein (RAP), an inhibitor of low-density lipoprotein receptor–related proteins (LRPs, including VLDL and APOER2; Herz et al., 1991; Bu and Schwartz 1998; Gong et al., 2007), was applied. The authors thus concluded that Reelin, acting via Dab1, is essential for the striking enrichment of HCN1 in the distal dendritic compartment of the pyramidal cells (Kupferman et al., 2014).

We have previously shown that the expression of Reelin in hippocampus is enhanced by 17β-estradiol (E2; Bender et al., 2010), the most potent estrogen, either synthesized in the gonads or locally produced and released as a neurosteroid from hippocampal neurons (Prange-Kiel et al., 2003; Kretz et al., 2004). We therefore reasoned that E2 should also influence the distribution of HCN1 channels in CA1, via the stimulation of Reelin expression. Here we tested this hypothesis using organotypic entorhino-hippocampal cultures to determine the effects of E2 on HCN1 distribution in CA1 and to examine whether these effects involve Reelin. Our data strongly suggest that E2 regulates HCN1 channel localization in CA1 pyramidal cells but argue against an involvement of Reelin. Data from an inducible conditional *reelin*-knockout mouse line (Lane-Donovan et al., 2015) further question an essential role of Reelin for the distal dendritic enrichment of HCN1 channels in pyramidal neurons, as was previously proposed (Kupferman et al., 2014).

## Material and Methods

### Organotypic cultures

Combined entorhino-hippocampal slice cultures, preserving perforant path connectivity, were used for these studies. Briefly, 5-d-old (P5) rats (Wistar, breeding stock from Charles River, RRID:RGD_737929) were decapitated; the hippocampus, with entorhinal cortex (EC) attached, was dissected out, gently placed on the platform of a McIlwain tissue chopper, and sliced perpendicular to the hippocampal longitudinal axis (400 μm). Slices were then transferred to preparation solution [minimal essential medium (MEM) supplemented with 2 mm glutamine, pH 7.3], but care was taken that slices adjacent to each other stayed attached. From these grouped slices, pairs or triples were isolated, of which each slice was transferred to a separate membrane insert (Millicell CM, 0.4-μm culture plate inserts, 30 mm diameter; Merck Millipore) and subjected to a different experimental condition (i.e., one slice of each pair or triple served as a vehicle-treated control, whereas the corresponding slices were subjected to experimental treatment). Cultures were maintained *in vitro* in a 37°C 95%/5% CO_2_ humidified incubator. Incubation medium consisted of 50% MEM, 25% Hanks’ balanced salt solution, and 25% heat-inactivated horse serum, supplemented with 2 mm glutamine, 30 mm glucose, 0.044% NaHCO_3_, 100 units/ml penicillin, and 100 μg/ml streptomycin (all tissue culture reagents were obtained from Invitrogen/Thermo Fisher Scientific). Medium was changed every second day. For immunohistochemistry, experimental treatment of the cultures usually started after 5 days *in vitro* (DIV) and lasted for 6 d (DIV5–DIV11), during which the medium of the experimental groups was supplemented with either E2 (100 nm, in H_2_O; Sigma, Cat# E4389), E2 (100 nm) + G36 (20 nm, in DMSO; Tocris, Cat# 4759), G1 (20 nm, in DMSO; Tocris, Cat# 3577), 4,4′,4″-(4-propyl-[1H]-pyrazole-1,3,5-triyl)*tris*phenol (PPT; 100 nm, in DMSO; Tocris, Cat# 1426), diarylpropionitrile (DPN; 100 nm, in DMSO; Tocris, Cat# 1495), receptor-associated protein-glutathione-S-transferase (GST-RAP, 10 μg/ml; see generation below), mouse anti-Reelin (CR-50) antibodies (2 μg/ml; MBL International, Cat# D223-3, RRID:AB_843523) or E2 (100 nm) + GST-RAP (10 μg/ml). For the controls, medium was supplemented with equivalent amounts of vehicle or suitable neutral molecules (GST or random mouse IgGs, respectively). In one experiment, cultures were supplemented with GST-RAP or GST (50 μg/ml) for only 48 h (DIV8–DIV10). Generally, at the end of treatment, cultures were fixed for 1 h with 4% paraformaldehyde (PFA) in PBS, then cryoprotected with 25% sucrose (2 h) and frozen on dry ice for immunohistochemistry (see below).

Additionally, the following experimental treatments were performed for Western blot analyses. (1) Cultures were incubated with E2 (100 nm), G1 (20 nm), or vehicle for 6 d (DIV5–DIV11), then harvested and deep-frozen for analysis of HCN1 and GPER1 expression levels. (2) On DIV10, cultures were incubated with GST-RAP or GST (10 μg/ml) for 24 h. On DIV11, 50 μl of Reelin-conditioned medium (see preparation below) were placed on top of each culture for 30 min. Slices were then harvested and immediately deep frozen in nitrogen for analysis of Dab1 protein expression (Dab1) and Dab1 phosphorylation (pDab1).

### Animals

For analyses *in vivo*, juvenile rats (Wistar, as above) with an equivalent age to the cultures (P16) and adult mice (P90–P100; C57BL/6J, breeding stock from Jackson Laboratory, RRID:IMSR_JAX:000664) were used. The animals were housed in the University Hamburg, Medical Center animal facility under controlled conditions and had access to food and water *ad libitum*. For immunohistochemistry, animals were deeply anesthetized using a ketamine-xylazine mixture (ketamine 12 mg/ml, xylazine 0.16% in saline, i.p.) and transcardially perfused with 4% PFA. Subsequently, the brains were removed from the skull, postfixed for 4 h in 4% PFA, cryoprotected with 25% sucrose for >48 h, and deep frozen in –50°C isopentane. From the adult female mice, the estrous cycle stage was determined postmortem using vaginal smears (Hoglund, 1972; Byers et al., 2012). All animal experiments were performed according to legal guidelines and were approved by the institutional committees for the care and use of laboratory animals (University Hamburg, Animal Care Committee, ORG 804). Additionally, male tamoxifen-inducible *reelin*-knockout mice (Reelin cKO) were examined. These mice were generated and held at the University of Texas, Southwestern Medical Center (Dallas, TX) by crossing an inducible Cre recombinase–expressing line (CAG-CreERT2) with mice in which the first exon of the *reelin*-gene was flanked with loxP sites (*Reln^flox/flox^*; Lane-Donovan et al., 2015). Control mice [wild type (WT)] were CAG-CreERT2-negative. At 2 months of age, both the Reelin cKO and the control mice received daily intraperitoneal injections for 5 d of tamoxifen (135 mg/kg; Sigma), dissolved in sunflower oil. Mice were perfused at 7–8 months of age, and brains were sent to the University of Hamburg for immunohistochemistry (IHC) analysis (see below).

### Immunohistochemistry

Brains and slice cultures were cut on a cryotome, and sections were collected in PBS. Subsequently, brain sections (25 μm) were processed “free-floating,” whereas slice culture sections (20 μm) were mounted to glass slides (sections from grouped cultures always on the same slide) and dried, before being processed. For IHC, both brain and culture sections were preincubated with 3% normal goat serum (in PBS) for 1 h at room temperature (RT), then primary antibodies were applied for 24 h at 4°C. The following primary antibodies were used: rabbit polyclonal anti-HCN1 (1:500; Merck Millipore, Cat# AB5884, RRID:AB_2115002), rabbit polyclonal anti-GPER1 (1:400; Abcam, Cat# ab39742, RRID:AB_1141090), mouse monoclonal anti-Reelin (1:500; Merck Millipore, Cat# MAB5366, RRID:AB_2285132), mouse monoclonal anti-tetratricopeptide repeat-containing Rab8b interacting protein (TRIP8b), constant region (1:1000; NeuroMab, clone N212/17, RRID:AB_10675453), rabbit polyclonal anti-aromatase (1:3000; Abcam, Cat# ab191093, RRID:AB_2737021; directed against amino acids 455-476 of rat aromatase), rabbit polyclonal anti-aromatase (1:200; BIOSS, Cat# bs-1292R, RRID:AB_10880885; directed against amino acids 65–120 of human aromatase), and rabbit polyclonal anti-aromatase (1:1200; directed against amino acids 488–502 of mouse aromatase; gift of Dr. I. Azcoitia, Madrid; Yague et al., 2006; RRID:AB_2631184). After primary antibody incubation, sections were washed twice in PBS, before secondary antibodies were applied for 3 h at RT: Alexa Fluor 488–coupled goat anti-rabbit IgGs (1:500; Invitrogen, Cat# A-11034, RRID:AB_2576217) or Alexa Fluor 546–coupled goat anti-mouse IgGs (1:500; Invitrogen, Cat# A-11030, RRID:AB_2534089). Sections were washed again and treated for 1 min with 4,6-diamidino-2-phenylindol (DAPI, Sigma). Free-floating sections were then mounted on glass slides. All sections were embedded with fluorescent mounting medium (Dako), coverslipped, and subsequently viewed and photographed using fluorescence microscopy (Keyence BZ9000: HCN1, GPER1, TRIP8b, Reelin) or confocal microscopy (Olympus FV1000: aromatase). To control for specificity, sections were processed according to the protocol above with primary antibodies omitted.

### Western blots

For protein expression analyses of HCN1, GPER1, total Dab1, and phosphorylated Dab1 (pDab1), tissue was homogenized in ice-cold RIPA lysis buffer (1% NP40, 0.1% SDS, 0.5% Na-deoxycholate, protease inhibitor, and phosSTOP; Roche). Lysates were cleared by centrifugation at 4°C and 13,000 × *g* for 30 min. From each sample, 30–50 µg was diluted in water and 5× Laemmli buffer (62.5 mm Tris, pH 6.8; 2% SDS; 10% glycerol; 5% 2-mercaptoethanol; 0001% bromophenol blue) to a final volume of 12.5 µl. The samples were heated to 95°C for 5 min and then immediately cooled on ice. Subsequently, samples from experimentally treated cultures were loaded side-by-side with the corresponding control cultures, then separated on a 10% polyacrylamide gel by gel electrophoresis (Invitrogen) in Laemmli running buffer (10% SDS, 3% Tris, 14% glycine) and transferred electrophoretically to polyvinylidene fluoride membranes with transfer buffer (0.02% SDS, 0015% Tris, 0.08% glycine). For blotting, the membranes were blocked with 5% bovine serum albumin (Dab1, pDab1) or milk powder (HCN1, GPER1) in PBS at RT for 1 h and incubated with primary antibodies: guinea pig polyclonal anti-HCN1 (1:500; Santa Cruz Biotechnology, Cat# sc-19706; this antibody is not available anymore; patterns were identical to those generated by the rabbit-anti-HCN1 that was used for IHC, see above), rabbit polyclonal anti-GPER1 (1:400), rabbit polyclonal anti-Dab1 (1:1000; Rockland Immunochemicals, Cat# 100-401-225, RRID:AB_2245755), or mouse monoclonal anti-phosphotyrosine (1:1000; Merck Millipore, clone 4G10, Cat# 05-321, RRID:AB_309678) in blocking solution at 4°C overnight. Mouse monoclonal anti-GAPDH (1:2000; Ambion/Thermo Fisher Scientific, Cat# AM4300, RRID:AB_437392) was co-applied for loading control. Secondary antibodies, conjugated with alkaline phosphatase, were applied for 1 h at RT (Western Breeze Chemiluminescent Immunodetection Kit, Invitrogen). The immunoreaction was visualized by enhanced chemiluminescence (FUSION-SL4 advanced imaging system; Vilber Lourmat Labtech).

### Generation of GST-RAP

The pGEX-kg vector was generated from the initial pGEX-2T vector (GE Healthcare) by cutting it with EcoR1 to insert a new linker. To generate the required pGEX-kg-RAP plasmid, cDNA (rat) of receptor-associated binding protein (RAP) was cloned via intersections EcoR1 and HindIII into the pGEX-kg vector (Herz et al., 1991). DH5α bacteria were transformed with the pGEX-kg-RAP with a heat shock at 41°C for 42 s followed by cooling down on ice. Bacteria were plated on ampicillin agar plates, which were incubated overnight at 37°C, then stored at 4°C. For subsequent procedure, liquid cultures were inoculated with transformed bacteria. RAP expression was induced by isopropyl-β-d-thiogalactopyranoside. After 5 h, bacteria were harvested by centrifugation. Cells were lysed with lysozyme and Triton X-100 and by mechanical stress. Proteins were stabilized with dithiothreitol. Extraction and purification were performed with glutathione-Sepharose columns. After elution of GST-RAP, protein concentration was determined by Bradford protein assay. Final protein concentrations were adjusted to 1 mg/ml, and aliquots were stored at –20°C.

### Preparation of Reelin-conditioned medium

HEK-293 cells were stably transfected with a plasmid containing full-length Reelin cDNA (D’Arcangelo et al., 1997). Serum-free supernatants containing secreted Reelin were collected as described by Förster et al. (2002).

### Analysis (IHC)

**Figure 1.**
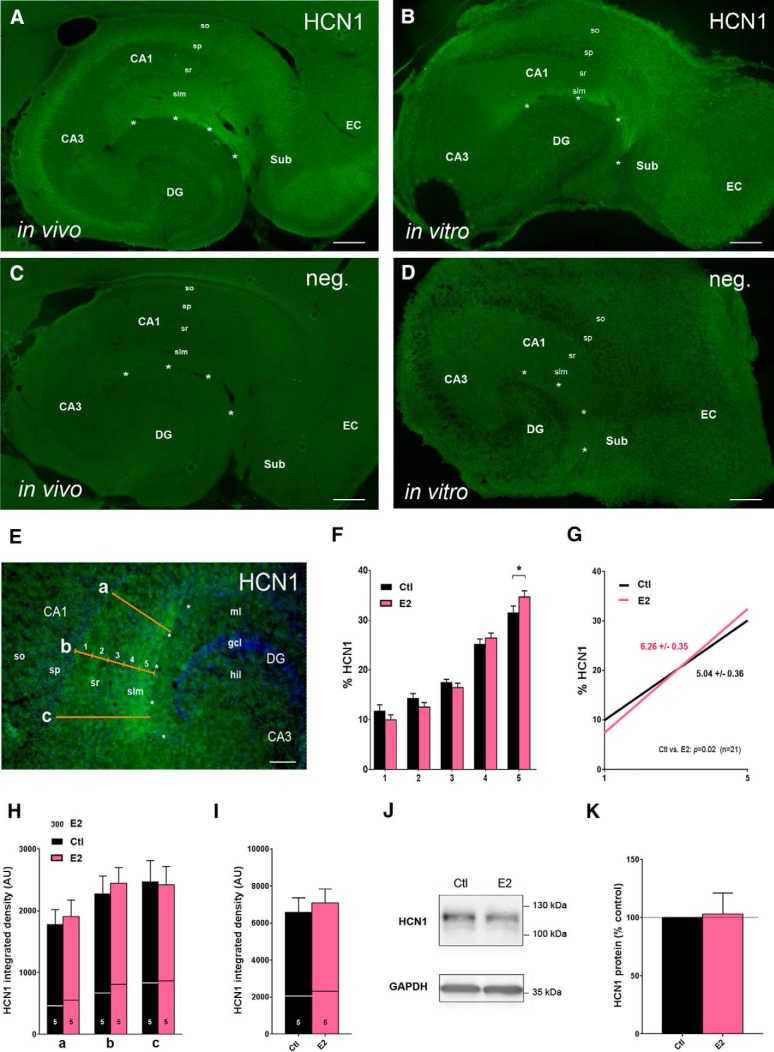
Estrogen (E2) promotes the distal dendritic enrichment of HCN1 in CA1. ***A***, ***B***, Comparison of HCN1 immunoreactivity (green) in a horizontal hippocampal section from a 16-d-old rat (*in vivo*, ***A***) and in a section from an entorhino-hippocampal organotypic slice culture prepared on postnatal day 5, and kept for 11 d *in vitro* (DIV11, ***B***). Note that the pattern *in vitro* reproduces the pattern *in vivo*, including HCN1 enrichment in distal CA1. ***C***, ***D***, No pattern was observed *in vivo* (***C***) and *in vitro* (***D***), when the primary antibody was omitted. ***E***, Scheme for the analysis of HCN1 distribution in CA1: three lines (positions *a*, *b*, *c*), connecting stratum pyramidale (sp) and the hippocampal fissure (asterisks) were subdivided into five equal segments, and relative HCN1 immunosignal (integrated line density) within each segment was determined. ***F***, ***G***, Results are illustrated as individual column bars (***F***) or linear regression analysis (slope) of the density increase from segment 1–5 (***G***). Note that E2 treatment from DIV5–11 increased the relative HCN1 immunosignal in segment 5 significantly (***F***) and resulted in a significantly steeper slope, indicating E2-induced distal dendritic enrichment of HCN1 (***G***). ***H***, ***I***, Comparison of the integrated density of HCN1 immunosignal (arbitrary units, AU) at analysis positions *a*, *b*, and *c* (***H***), or summarized for all positions (***I***) did not reveal differences of total HCN1 immunosignal between E2- and vehicle-treated cultures at any of the positions chosen for analysis. Horizontal lines demarcate the proportion of HCN1 signal in segment 5 at each position (*a*: 26% ± 1% vs. 31% ± 2%, *b*: 30% ± 2% vs. 33% ± 2%, *c*: 34% ± 3% vs. 36% ± 2%, in vehicle- and E2-treated cultures, respectively). ***J***, Representative Western blots for HCN1 (upper lane) or GAPDH for loading control (lower lane), using tissue from cultures treated with either vehicle (left bands, Ctl) or E2 (right bands): no E2-effect on total HCN1 protein expression within the cultures was evident. Note the characteristic double bands for HCN1, at 105 and 125 kDa, indicative of differential glycosylation of HCN1 (Zha et al., 2008). Data are quantified in ***K***. Scale bars: 250 μm (***A–D***), 100 μm (***E***). CA, cornu ammonis; DG, dentate gyrus, EC, entorhinal cortex; hil, hilus; gcl, granule cell layer; ml, molecular layer; so, stratum oriens, sr, stratum radiatum; Sub, subiculum. The section in ***C*** has been counterstained with DAPI (blue).

For quantitative analysis of HCN1 distribution in slice cultures, sections from grouped cultures (pairs or triples, see above) were mounted to the same slide and immunostained for HCN1. Subsequently, CA1 was photographed at 100× magnification using identical illumination for each section by a photographer who was blinded to the experimental condition. For further analysis (also blinded), one picture per culture was chosen. Using Fiji (ImageJ) software (National Institutes of Health), three lines were then drawn in CA1, from the pyramidal cell layer to the hippocampal fissure, at defined positions (illustrated in Fig. 1*E*): (a) above the crest of the granule cell layer, (b) above the midpoint between crest and tip of the granule cell layer, and (c) above the tip of the granule cell layer. Along the proximal-distal axis, these lines were subdivided into five equal segments (1–5), with segment 1 adjoining the pyramidal cell layer (most proximal) and segment 5 adjoining the hippocampal fissure (most distal). Integrated line density (indicated as arbitrary units, AU) was determined along these lines using NIH Fiji software. Background signal, determined in the molecular layer of the dentate gyrus, was subtracted. Subsequently, the proportion of HCN1 signal per segment (percent of total HCN1 AU) was calculated at each of the three positions. Data from the three positions were then averaged to obtain one representative value for each segment per analyzed culture (each culture equaling *n* = 1). The illustrated scheme (Fig. 1*E*) was also used for the analysis of HCN1 distribution in Reelin cKO versus WT mice (*n* = 5, each) and in adult male and female mice for the analysis of estrus cycle effects (*n* = 6, each group). For these analyses, three topically defined coronal sections (corresponding to positions 71, 74, and 77 of the Allen Mouse Brain Atlas, http://mouse.brain-map.org/static/atlas) were selected per animal, and left and right hippocampus was analyzed in each section, resulting in a total of six values per segment, from which the mean was calculated (denoting one representative value per animal, equaling *n* = 1). In addition, from the adult female mice (*n* = 6; 3 in proestrus, 3 in diestrus) another set of sections (as above) was chosen to quantify aromatase immunosignal in CA1 stratum lacunosum-moleculare (slm) relative to expression in stratum radiatum (sr). For this purpose, sections were immunostained with rabbit polyclonal anti-aromatase (Abcam, see above). CA1 was then photographed at 100× magnification with confocal microscopy, and the integrated density of aromatase immunosignal was determined, both in sr and slm, at three defined positions above the granule cell layer (positions *a*, *b*, and *c*; as above) using NIH Fiji software. Subsequently, the mean integrated density for each layer was determined, and density in slm was compared to density in the corresponding sr for each animal. Data are presented as aromatase in slm/sr (%).

### Quantitative Western blots

For quantitative analyses of HCN1, GPER1, Dab1, and pDab1 in organotypic slices, all cultures deriving from one pup that had been subjected to the same treatment (usually 4–7) were pooled, and tissue was prepared for Western blotting as described above (thus, each pup equaling *n* = 1). Signal intensity of bands was quantified by densitometry, using NIH Fiji software and GAPDH for loading control. Subsequently, data from the experimentally treated cultures were compared to the data from the corresponding controls (running side by side) and presented as percent of control.

### Statistics

Relative HCN1 distribution (percent of total HCN1) in each of the five segments, resulting from integrated line density measurements, is illustrated in column bar graphs. Two types of statistical tests were used to analyze the data: (1) Wilcoxon matched pairs signed rank test (if two experimental groups were compared) or Friedman test, followed by Dunn’s multiple comparison test (if three groups were compared) were applied to test for differences within individual segments, if data were paired and nonparametric (i.e., from slice cultures). For unpaired data, such as those resulting from analyses in mice, Mann–Whitney test (two groups: Reelin cKO versus WT) or Kruskal–Wallis test, followed by Dunn’s multiple comparison test [three groups: estrus cycle analysis (male versus proestrus and diestrus females)] were used. (2) In addition, to pinpoint comprehensive trends, a comparison of the slopes of the gradients from segments 1–5 was performed using linear regression analysis, a method equivalent to the analysis of covariance (ANCOVA). For other analyses, paired *t* test [e.g., total HCN1 (AU) along lines a, b, c; see Fig. 1*H*,*I*] or Wilcoxon signed rank test (for Western blot analyses, comparing experimentally treated and corresponding vehicle-treated control groups, set to 100%; e.g., Fig. 1*K*) were applied. Statistical analyses were performed with Prism software (Prism 6, GraphPad). Data are presented as mean ± standard error of the mean.

## Results

### E2 promotes the distal dendritic enrichment of HCN1 in CA1 pyramidal cells

**Figure 2.**
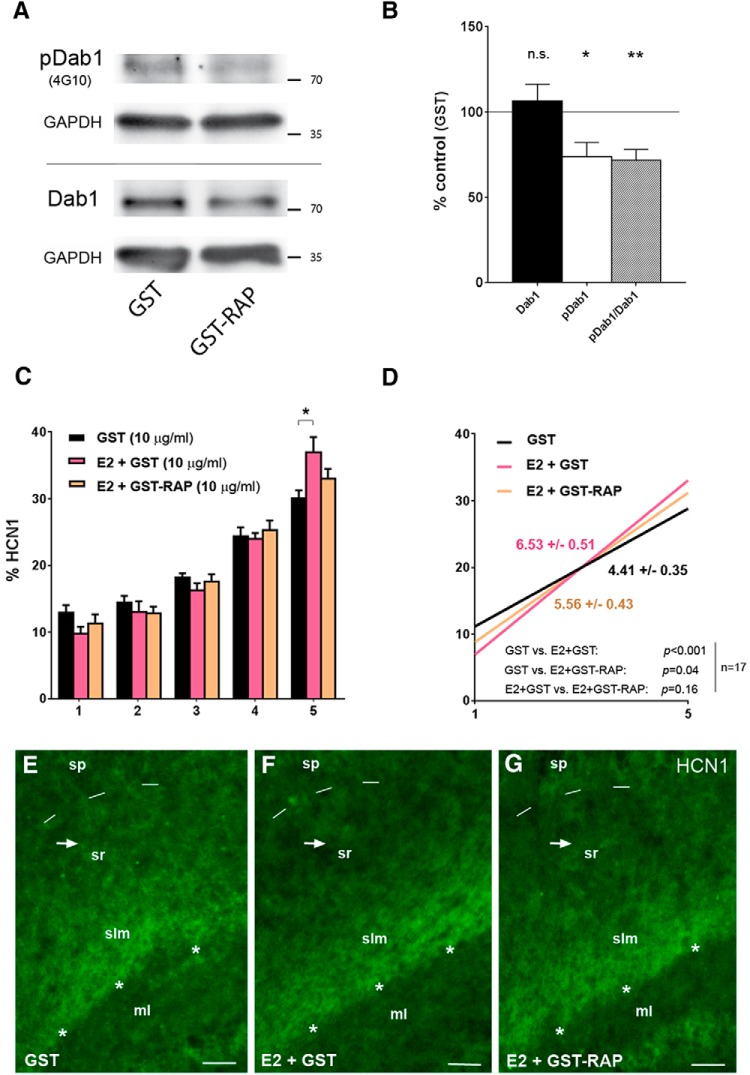
Coapplication of GST-RAP does not prevent the E2-induced distal dendritic HCN1 enrichment *in vitro*. ***A***, Western blots showing phosphorylated Dab1 (pDab1, upper bands, detected by anti-phosphotyrosine antibody 4G10) and total Dab1 (lower bands) in “paired” slice cultures that were treated for 24 h (DIV10–11) with either GST-RAP or GST (10 μg/ml each) and were exposed to Reelin-conditioned medium for 30 min before harvesting. GAPDH was used for loading control. ***B***, Quantitative analysis of Dab1 and pDab1 levels (relative to GAPDH) revealed that Reelin-induced pDab1 was reduced in the GST-RAP-treated slices, while total Dab1 was not significantly different compared to the GST-treated controls. Thus, pDab1/Dab1 was reduced to 72% ± 6% of control levels after 24-h GST-RAP treatment (*p* < 0.01; *n* = 15). ***C***, ***D***, E2 (+GST)-treatment (pink) of cultures for 6 d (DIV5–11) caused a significant HCN1 accumulation in segment 5 (***C***) and a significantly increased slope (***D***) compared with controls (GST, black) that was not efficiently reduced, if GST-RAP (10 μg/ml) was coapplied (orange; *n* = 17, each group). ***E–G***, Representative photographs from a culture “triple,” of which one culture served as a vehicle (GST)-treated control (***E***), while the others were treated with either E2 + GST (***F***) or E2 + GST-RAP (***G***). Note that HCN1 is accumulated at the hippocampal fissure (asterisks) at all conditions, but if E2 was present (***F***, ***G***), less HCN1 immunosignal is visible in stratum radiatum (sr, arrows), indicating (relative) HCN1 enrichment in stratum lacunosum-moleculare (slm). Scale bars: 80 μm (***E–G***). Dashed lines demarcate the border of stratum pyramidale (sp). ml, molecular layer.

We initially studied effects of E2 on HCN1 distribution in CA1 using combined entorhino-hippocampal slice cultures, prepared from early postnatal (P5) rats and cultivated for a total of 11 d (DIV11). Experimental treatment (DIV5–DIV11) specifically comprised the time span during which the HCN1 pattern in CA1 is developmentally forming (Brewster et al., 2007; Shin and Chetkovich, 2007). As shown in Fig. 1, the characteristic *in vivo* pattern of HCN1 distribution in CA1 is reproduced *in vitro* at DIV11 (compare Fig. 1*A*,*B*): HCN1 immunohistochemistry increases from proximal to distal, resulting in an enrichment at the hippocampal fissure (indicated by asterisks). No pattern was observed if the primary antibody was omitted (Fig. 1*C*,*D*). Quantitative analysis along proximal-distal lines *in vitro* (Fig. 1*E*) revealed that, under control conditions, 57 ± 2% of the HCN1 immunosignal located to segments 4 and 5, which roughly comprise stratum lacunosum-moleculare (slm), with the majority (31 ± 1%) localizing to segment 5, which borders the hippocampal fissure (Fig. 1*F*). Supplementing the culture medium with E2 (10^−7^ m) augmented the proportion of HCN1 channels localizing to segments 4 and 5, which was particularly evident in segment 5 (34 ± 1%, *n* = 21; Wilcoxon matched pairs signed rank test: *p* = 0.02; Figs. 1*F*,*H*,*I*). Correspondingly, the proportion of channels localizing to segments 1–3, comprising stratum radiatum (sr), decreased significantly (E2: 39 ± 2% vs. Ctl: 43 ± 2%, *n* = 21; Wilcoxon matched pairs signed rank test: *p* = 0.04). Consequently, the proximal-distal HCN1 gradient, denoted by the slope from segment 1–5, was significantly increased in the presence of E2 (E2: 6.26 ± 0.35 vs. Ctl: 5.04 ± 0.36, *n* = 21; linear regression analysis: *F* = 5.9, *DFn* = 1, *DFd* = 206, *p* = 0.016), showing that E2 had promoted the developmental enrichment of HCN1 in the CA1 distal dendritic segment (Fig. 1*G*). These initial findings were reliably reproduced in all follow-up experiments, in which an E2-treated group of cultures was included (e.g., Figs. 2*C*,*D*), resulting in a total of 52 cultures that were analyzed for the effect of E2. Among these 52 cultures, no sex bias was observed [in segment 5, female: 36 ± 2% (E2) vs. 31 ± 1% (Ctl), *n* = 27; Wilcoxon matched pairs signed rank test: *p* = 0.004; male: 35 ± 1% (E2) vs. 31 ± 1% (Ctl), *n* = 25; Wilcoxon matched pairs signed rank test: *p* = 0.02]. Therefore, the sex of the animals from which the cultures were generated was not further considered in subsequent experiments. Differences between experimental groups were not evident for total HCN1 signal intensity in CA1 (E2: 7078 ± 767 vs. Ctl: 6570 ± 789 AU, *n* = 21; paired *t* test: *p* = 0.6; Fig. 1*I*) and HCN1 protein levels in the whole cultures (E2: 103 ± 18% relative to controls, *n* = 9; Wilcoxon signed rank test: *p* = 0.6; Fig. 1*J*,*K*), suggesting that it is a distal dendritic shift of the HCN1 channels, involving altered channel trafficking, rather than altered HCN1 expression, that causes the increased HCN1 immunosignal in the distal segments.

### E2-induced distal enrichment of HCN1 in CA1 does not involve Reelin signaling

We next questioned whether the effect of E2 on HCN1 distribution in CA1 is mediated by Reelin. For this purpose, we used the LRP inhibitor GST-RAP at concentrations (10 μg/ml) that had previously been shown to reduce Reelin signaling in neuron cultures (Groc et al., 2007) and reduced Reelin-induced Dab1-phosphorylation (pDab1/Dab1) to 72% ± 6% of control levels (GST) in slice cultures, which were preincubated with GST-RAP for 24 h (DIV10–DIV11) and then exposed to Reelin (*n* = 15; Wilcoxon signed rank test: *p* = 0.001; Fig. 2*A*,*B*). However, co-applying E2 and GST-RAP for a 6-d treatment period (DIV5–DIV11, as above) did not efficiently block the distal enrichment of HCN1 that was induced in CA1, if E2 was supplemented only with GST [segment 5: E2 + GST: 37% ± 2% vs. GST alone: 30% ± 1%; Friedman test: *p* = 0.07; Dunn’s multiple comparisons test: *p* < 0.05 (E2 + GST vs. GST); slopes: E2 + GST: 6.53 ± 0.52 vs. GST: 4.41 ± 0.35, *n* = 17; linear regression analysis: *F* = 11.5, *DFn* = 1, *DFd* = 166, *p* < 0.001; Fig. 2*C–G*]. In fact, co-application of E2 and GST-RAP still resulted in a significantly steeper slope relative to the controls (E2 + GST-RAP: 5.56 ± 0.43 vs. GST: 4.41 ± 0.35, *n* = 17; linear regression analysis: *F* = 4.5, *DFn* = 1, *DFd* = 166, *p* = 0.04; Fig. 2*C–G*), whereas no significant difference was evident if E2 + GST and E2 + GST-RAP slopes were compared (linear regression analysis: *F* = 2.0, *DFn* = 1, *DFd* = 166, *p* = 0.16; Fig. 2*C–G*). We therefore reasoned that the GST-RAP–induced inhibition of Reelin signaling was either not sufficient to suppress the E2-induced increase of HCN1 in distal CA1 or, more generally, that Reelin is not required for the distal HCN1 enrichment in our experimental setting.

**Figure 3.**
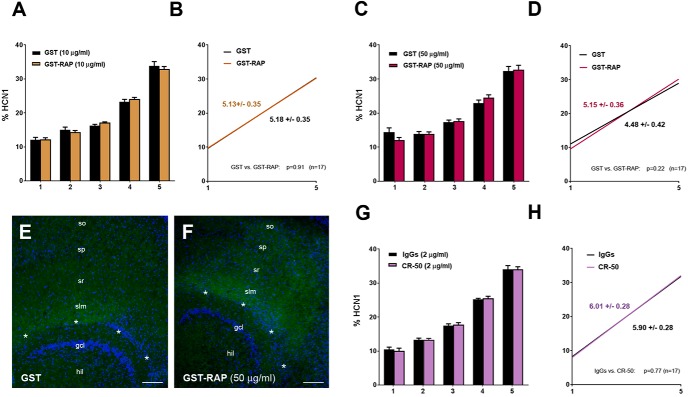
Reelin is not required for HCN1 distal dendritic enrichment in CA1 *in vitro*. ***A–D***, To test whether Reelin is generally required for the distal dendritic enrichment of HCN1 in CA1 *in vitro*, organotypic slice cultures were treated with GST or GST-RAP for 6 days (10 μg/ml, DIV5–11, *n* = 17; ***A***, ***B***) or in higher concentration for 48 h (50 μg/ml, DIV8–10, *n* = 17; ***C***, ***D***). None of these treatments prevented HCN1 enrichment in distal CA1. ***E***, ***F***, Photographs show HCN1 distribution in slice cultures treated for 48 h with either GST (50 μg/ml; ***E***) or GST-RAP (50 μg/ml; ***F***), demonstrating unaltered HCN1 enrichment in distal CA1. Sections have been immunostained for HCN1 (green) and counterstained with DAPI (blue). ***G***, ***H***, Supplementing Reelin-blocking CR-50 antibodies (2 μg/ml, DIV5–11, *n* = 17) in the culture medium did also not alter HCN1 distribution in CA1. Note: none of the treatments above altered total HCN1 expression in CA1 (see Results). Scale bars (***E***, ***F***): 100 μm. hil, hilus; gcl, granule cell layer; slm, stratum lacunosum-moleculare; sp, stratum pyramidale; so, stratum oriens; sr, stratum radiatum. Asterisks indicate the hippocampal fissure.

To address the latter possibility, GST-RAP (or GST) was applied to the culture medium of P5 cultures without co-applying E2 for 6 d (10 μg/ml, DIV5–DIV11; Fig. 3*A*,*B*) or 48 h (50 μg/ml, DIV8–DIV10; Fig. 3*C–F*), thus reproducing the experimental conditions of Kupferman et al. (2014). However, none of these treatments altered the somatodendritic gradient of HCN1. Even the high concentrations of GST-RAP (50 μg/ml) did not prevent the accumulation of HCN1 at the hippocampal fissure [slopes: 5.15 ± 0.36 (GST-RAP) vs. 4.48 ± 0.42 (GST), *n* = 17; linear regression analysis: *F* = 1.5, *DFn* = 1, *DFd* = 166, *p* = 0.22; Fig. 3*D*]. Similarly, Reelin-binding antibody CR-50 (2 μg/ml), applied to the cultures for 6 d (DIV5–DIV11), did not affect HCN1 distribution in CA1 [slopes: 6.01 ± 0.28 (CR-50) vs. 5.90 ± 0.28 (IgG), *n* = 17; linear regression analysis: *F* = 0.09, *DFn* = 1, *DFd* = 166, *p* = 0.77; Fig. 3*G*,*H*]. None of these treatments altered total HCN1 expression in CA1, as total integrated densities were not significantly different from controls in the experimental groups [GST-RAP (10 μg/ml): 1859 ± 197 vs. GST (10 μg/ml): 1620 ± 183 AU; GST-RAP (50 μg/ml): 2502 ± 282 vs. GST (50 μg/ml): 2182 ± 244 AU; CR-50: 2615 ± 366 vs. IgG: 2905 ± 258 AU; paired *t* tests: *p* = 0.24, 0.32 and 0.50, respectively].

**Figure 4.**
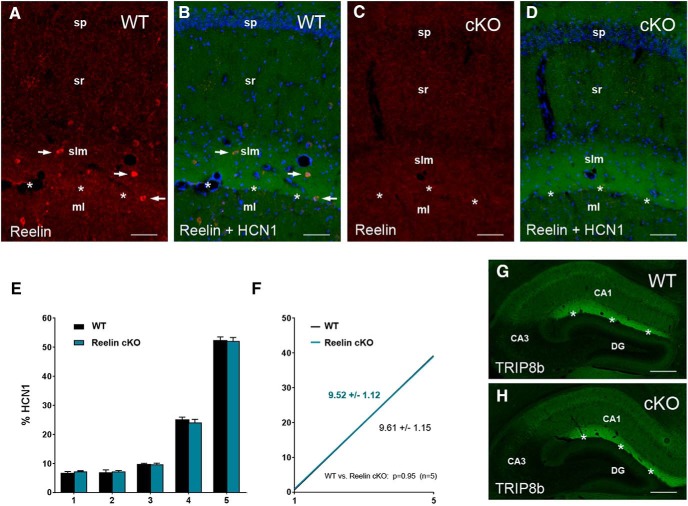
Reelin is not required for HCN1 distal dendritic enrichment in CA1 *in vivo*. ***A–D***, Photographs show CA1 in adult mice (7–8 months), in which *reelin* was deleted at 2 months of age via tamoxifen induction (cKO; ***C***, ***D***), or the corresponding wild types (WT; ***A***, ***B***). Sections have been immunostained for Reelin (red; ***A–D***) and HCN1 (green; ***B*** and ***D***). Note that Reelin-expressing cells, which are prominent in stratum lacunosum-moleculare (slm) of the WT (see arrows in ***A*** and ***B***), are completely absent in the cKO-mice (***C***, ***D***). This did not prevent the accumulation of HCN1 in distal CA1 near the hippocampal fissure (indicated by asterisks). ***E***, ***F***, Quantification revealed a virtually identical somatodendritic HCN1 gradient in Reelin cKO compared with WT mice, both within individual segments (***E***; Mann–Whitney test: *p* > 0.05 for each segment; *n* = 5 each group) and if the slopes were compared (***F***; linear regression analysis: *p* = 0.95). ***G***, ***H***, Similarly, TRIP8b enrichment in distal CA1 was not altered in Reelin-deficient mice (***H***) compared to WT (***G***). Scale bars: 80 μm (***A–D***), 400 μm (***G***, ***H***). CA, cornu ammonis; DG, dentate gyrus; ml, molecular layer; sp, stratum pyramidale; sr, stratum radiatum.

Because different experimental conditions could have caused the discrepancies with Kupferman et al. (2014), we further examined the distribution of HCN1 in 7–8-month-old tamoxifen-inducible *reelin*-knockout mice, in which the *reelin* gene was eliminated at 2 months of age (Reelin cKO; Lane-Donovan et al., 2015). In these mice, the hippocampus had a normal cytoarchitecture (e.g., cellular layers were distinct and the hippocampal fissure was clearly recognizable), but Reelin-expressing cells were completely absent (compare Fig. 4*A*,*C*). Nevertheless, HCN1 distribution in CA1 was not different from that in control mice (WT; compare Fig. 4*B*,*D*), and no effect of Reelin deficiency on the distal dendritic enrichment of HCN1 was detectable [slopes: 9.61 ± 1.15 (WT) vs. 9.52 ± 1.12 (Reelin cKO), *n* = 5 each; linear regression analysis: *F* = 0.003, *DFn* = 1, *DFd* = 46, *p* = 0.95; Fig. 4*E*,*F*]. Furthermore, the distribution of TRIP8b, an HCN channel auxiliary subunit that is important for HCN1 subcellular trafficking (Lewis et al., 2011; Piskorowski et al., 2011; Wilkars et al., 2012), was virtually unaffected by Reelin deficiency (Fig. 4*G*,*H*; but see Kupferman et al., 2014). Taken together, these findings argue against a critical role of Reelin in the regulation of HCN1 distribution in CA1.

### E2-induced distal enrichment of HCN1 in CA1 is mediated via GPER1

**Figure 5.**
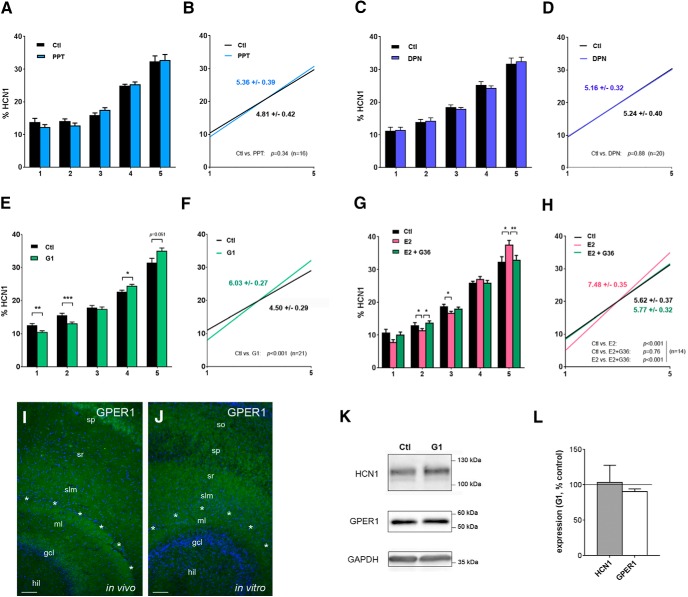
E2 effects on HCN1 distribution in CA1 are mediated by GPER1. ***A–F***, Application of agonists for estrogen receptors ERα (PPT, 100 nM, *n* = 16; ***A***, ***B***), ERβ (DPN, 100 nM, *n* = 20; ***C***, ***D***), or GPER1 (G1, 20 nM, *n* = 21; ***E***, ***F***) to the culture medium for 6 d (DIV5–11) resulted only for G1 in a significant enhancement of HCN1 distal dendritic expression (***E***, ***F***). No effect was observed with PPT or DPN. ***G***, ***H***, Coapplication of E2 (100 nM) with the GPER1 antagonist G36 (20 nM, *n* = 14) abolished the E2-mediated HCN1 enrichment in distal CA1, suggesting that the G protein–coupled receptor GPER1 is mediating the E2 effect. ***I***, ***J***, Photographs show CA1 in sections from a P16 rat (*in vivo*, ***I***) and from an age-equivalent slice culture (*in vitro*; P5, DIV11; ***J***). Sections have been immunostained for GPER1 (green) and counterstained with DAPI (blue). Note: both *in vivo* and *in vitro*, robust GPER1-immunosignal is detectable in the areas surrounding the hippocampal fissure (asterisks) and in the CA1 dendritic field, but signal is almost absent in the hilus (hil). ***K***, Representative Western blots showing HCN1 (upper lane), GPER1 (middle lane), and GAPDH bands (lower lane, used as loading control) in tissue from slice cultures treated for 6 d (DIV5–11) with either G1 or vehicle (Ctl). ***L***, Quantification did not reveal any effect of G1 treatment on either HCN1 (103% ± 24%, *p* = 0.9, *n* = 6) or GPER1 (91% ± 4%, *p* = 0.09, *n* = 6) protein expression in the cultures. Scale bars (***I***, ***J***): 100 μm. gcl, granule cell layer; slm, stratum lacunosum-moleculare; sp, stratum pyramidale; so, stratum oriens; sr, stratum radiatum.

We next wanted to understand how E2 effects on HCN1 distribution in CA1 are mediated, if Reelin is not involved. For this purpose, selective agonists of the estrogen receptors ERα (PPT, Fig. 5*A*,*B*), ERβ (DPN, Fig. 5*C*,*D*), and G protein–coupled GPER1 (G1, Fig. 5*E*,*F*) were applied for 6 d (as above) to the culture medium, and effects on HCN1 distribution in CA1 were determined. Only treatment with G1 caused a significant shift of HCN1 toward the distal segments and a significant enhancement of the slope (G1: 6.03 ± 0.27 vs. Ctl: 4.50 ± 0.29, *n* = 21, linear regression analysis: *F* = 14.5, *DFn* = 1, *DFd* = 206, *p* < 0.001, Fig. 5*F*), whereas none of the other agonists had an effect (PPT: 5.36 ± 0.39 vs. Ctl: 4.81 ± 0.42, *n* = 16, *F* = 0.9, *DFn* = 1, *DFd* = 156, *p* = 0.34, Fig. 5*B*; DPN: 5.16 ± 0.32 vs. Ctl: 5.24 ± 0.40, *n* = 20, *F* = 0.02, *DFn* = 1, *DFd* = 196, *p* = 0.88, Fig. 5*D*), suggesting that E2 effects are mediated via GPER1. To control for this scenario, we co-applied E2 (100 nm) with the GPER1 antagonist G36 (20 nm; Figs. 5*G*,*H*) during the 6-d treatment period and found that the E2-induced distal dendritic enrichment was abolished in the presence of G36, as evident from the bar charts [e.g., segment 5: 37 ± 1% (E2) vs. 32 ± 1% (Ctl) and 33 ± 2% (E2 + G36), *n* = 14 each; Friedman test: *p* = 0.004; Dunn’s multiple comparisons test: *p* < 0.05 (E2 vs. Ctl), *p* < 0.01 (E2 vs. E2 + G36), *p* > 0.05 (Ctl vs. E2 + G36), Fig. 5*G*] and the slopes [5.62 ± 0.37 (Ctl) vs. 7.48 ± 0.35 (E2) and 5.77 ± 0.32 (E2 + G36); linear regression analyses: *F* = 13.4, *DFn* = 1, *DFd* = 136, *p* < 0.001 (E2 vs. Ctl); *F* = 16.8, *DFn* = 2, *DFd* = 159, *p* < 0.001 (E2 vs. E2 + G36); *F* = 0.1, *DFn* = 1, *DFd* = 136, *p* = 0.76 (Ctl v. E2 + G36), Fig. 5*H*]. Further, GPER1 immunohistochemistry suggests that this receptor is in a suitable position to mediate site-specific E2 effects in CA1, as both in P16 rats and in age-equivalent cultures, GPER1 expression is high in the CA1 dendritic field, but very low in other areas, such as the hilus (Figs. 5*I*,*J*; see also Waters et al., 2015). Notably, G1 treatment did not significantly alter total HCN1 or GPER1 protein levels within the cultures [relative to control: 103 ± 24% (HCN1), 91 ± 4% (GPER1), *n* = 6 each; Wilcoxon matched pairs signed rank test: *p* = 0.9 and *p* = 0.09, respectively; Fig. 5*K*,*L*].

### Distal dendritic enrichment of HCN1 is not affected by the female estrous cycle

**Figure 6.**
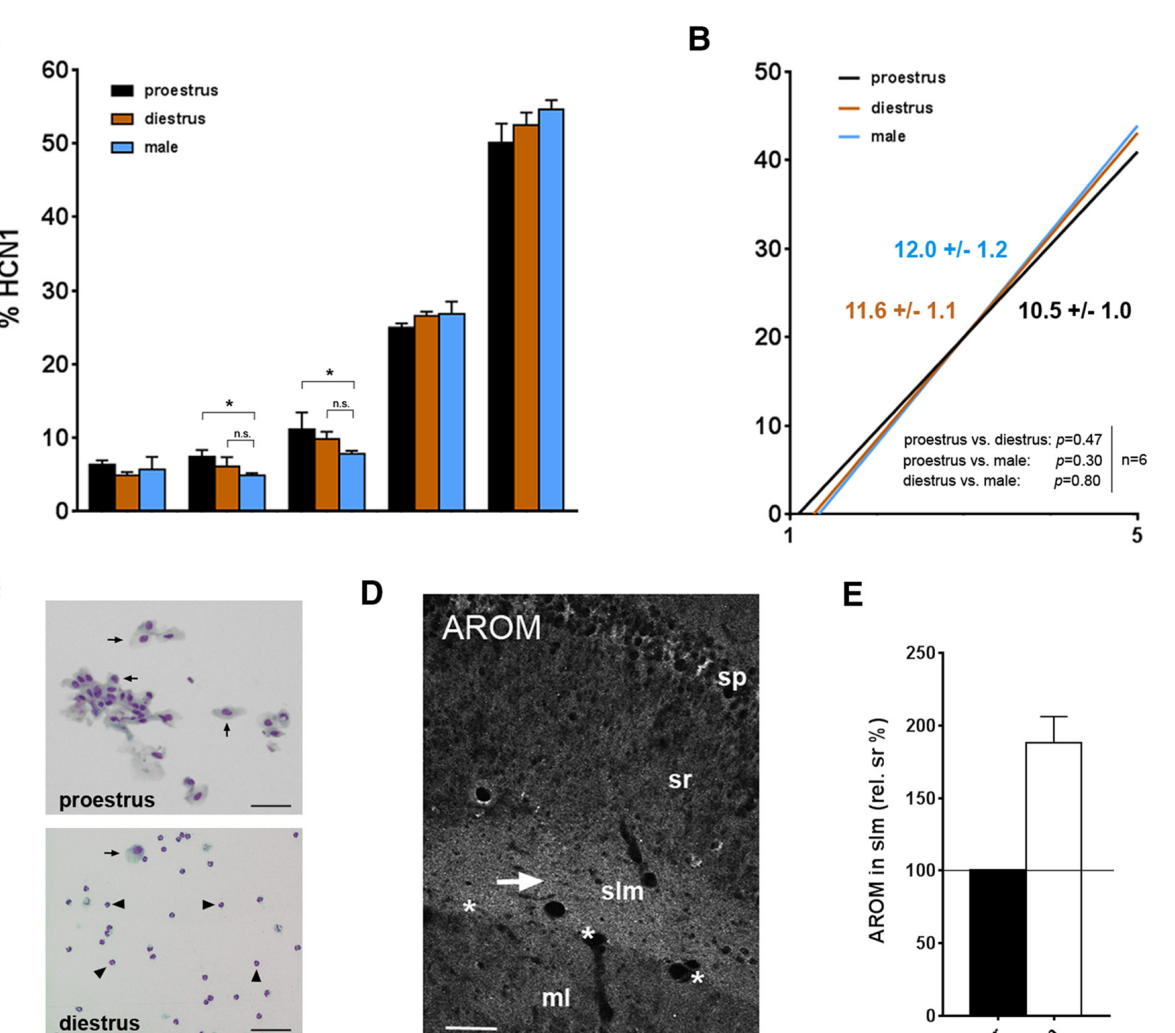
Effects of the female estrous cycle. ***A***, ***B***, Quantitative analysis of CA1 HCN1 distribution in sections from female mice, which were in stages of the estrous cycle with the most discrepant E2 serum levels (black: high E2 in proestrus, orange: low E2 in diestrus; *n* = 6 each), and from age-equivalent male mice (blue, *n* = 6) for comparison. No significant differences between females in differing cycle stages were evident within individual segments (***A***) or between the slopes (***B***). When males and females were compared, a trend toward reduced HCN1 in stratum radiatum was apparent in the males (relative to proestrus females) in individual segments [e.g., in segment 2: Kruskal–Wallis test: *p* = 0.04; Dunn’s multiple comparisons test: *p* < 0.05 (male vs. proestrus), ***A***], but this trend did not prove to be significant, if linear regression analysis was applied (***B***). ***C***, Representative cytology showing vaginal smears deriving either from a female mouse in proestrus (upper panel) or a mouse in diestrus (lower panel). Note that in proestrus almost exclusively nucleated epithelial cells (arrows) are observed in the vaginal smear. In diestrus, however, leukocytes (arrowheads) predominate, whereas nucleated epithelial cells are rarely seen (Byers et al., 2012). ***D***, Confocal microscopy illustrating aromatase (AROM) expression in the CA1 dendritic field, specifically in stratum lacunosum-moleculare (slm, arrow). Because neuronal aromatase activity is inversely related to serum E2 levels, due to its regulation by the hypothalamic-pituitary axis (Prange-Kiel et al., 2008; Kato et al., 2013), it could balance the varying levels of peripheral hormones during the estrous cycle. Note: to control for specificity, aromatase immunohistochemistry was performed with three different antibodies directed against three different antigenic regions (see Methods for details). All revealed virtually identical staining patterns, which were not observed if primary antibodies were omitted. ***E***, Quantification of relative aromatase expression indicates a significantly higher expression in slm (188% ± 18%, *p* = 0.03, *n* = 6) compared to stratum radiatum (sr). Scale bars: 50 μm (***C***), 80 μm (***D***). sp, stratum pyramidale; ml, molecular layer. Asterisks indicate the hippocampal fissure.

The organotypic slice culture model is most suitable to address developmental processes, including the maturation of the HCN1 distribution pattern, which is evolving during the first postnatal weeks (Brewster et al., 2007; Shin and Chetkovich, 2007). However, a specific feature of estrogens is their fluctuation during the female estrous cycle, and several cycle-dependent effects on hippocampal plasticity have been described (Woolley and McEwen, 1992; Scharfman and MacLusky, 2006). We therefore wanted to know whether the localization of the HCN1 channels in CA1 changes during the estrous cycle. For this purpose, HCN1 distribution in CA1 was analyzed in female mice that were either in proestrus (high serum E2; Fig. 6*C*, upper panel) or diestrus (low serum E2, Fig. 6*C*, lower panel). Age-equivalent male mice (*n* = 6) were examined for comparison. The analysis revealed no significant differences between the female cycle stages [slopes: 10.5 ± 1.0 (proestrus) vs. 11.6 ± 1.1 (diestrus), *n* = 6 each; linear regression analysis: *F* = 0.5, *DFn* = 1, *DFd* = 56, *p* = 0.47; Fig. 6*A*,*B*]. When males and females were compared, differences relative to proestrus females became apparent in some segments (segments 2 and 3; Fig. 6*A*). However, when the slopes were considered, the HCN1 gradient in males proved not to be significantly different from the gradients in proestrus or diestrus females (male slope: 12.0 ± 1.2, *n* = 6; linear regression analysis: *F* = 0.9, *DFn* = 1, *DFd* = 56, *p* = 0.35 relative to proestrus; *F* = 0.07, *DFn* = 1, *DFd* = 56, *p* = 0.8 relative to diestrus; Fig. 6*B*). This would argue against a role of gonadally synthesized estradiol. However, because under physiologic conditions hippocampus-derived E2 is generated in both male and female rodent hippocampus (Vierk et al., 2014; Hojo and Kawato, 2018), hippocampal aromatase could play a regulatory role (Prange-Kiel et al., 2003; Hojo et al., 2004). Indeed, as indicated by immunohistochemistry, aromatase expression is substantial in the dendritic area of CA1 (Fig. 6*D*) and most prominent in stratum lacunosum-moleculare [188 ± 18%, compared to expression in stratum radiatum, *n* = 6 (3 proestrus, 3 estrus); Wilcoxon signed rank test: *p* = 0.03; Fig. 6*E*].

## Discussion

The apical dendrites of CA1 pyramidal cells integrate excitatory synaptic input from mainly two sources: from CA3 via the Schaffer collaterals, which terminate proximally in stratum radiatum, and from the entorhinal cortex via the perforant path, which forms synapses distally in stratum lacunosum-moleculare. To achieve proper integration, different sets of ion channels localize to the proximal and distal dendritic portions, enabling these compartments to shape the computational and storage capabilities of neurons according to functional requirements (Magee and Johnston, 2005; Nusser, 2012). Little is known yet about the mechanisms that direct the ion channels to their specific dendritic compartments. Here we provide evidence that 17β-estradiol (E2), acting through the G protein–coupled estrogen receptor GPER1, is a regulating factor, as it promotes the enrichment of HCN1 channels within the distal segment of CA1 pyramidal cell apical dendrites. We could not verify, however, the previously suggested role of Reelin for the establishment and preservation of the somatodendritic gradient of HCN1 channels in CA1 (Kupferman et al., 2014).

The findings by Kupferman et al. (2014), suggesting that disrupting Reelin signaling causes mislocalization of HCN1 and its auxiliary subunit TRIP8b in hippocampal and neocortical pyramidal neurons, were intriguing, because they provided for the first time a hint at a non–cell-autonomous factor that regulates HCN channel distribution. The primary source of Reelin are Cajal-Retzius (CR) cells, which are considered pioneer neurons required for cortical lamination (D’Arcangelo et al., 1995; Ogawa et al., 1995; Tissir and Goffinet, 2003; Förster et al., 2006). Most CR cells die early during development. However, in hippocampus, a substantial number of them survive and continue to produce Reelin throughout maturity (Anstötz et al., 2016). These cells, bordering the hippocampal fissure (see Fig. 4*A*), are generally well positioned to promote HCN1 trafficking toward the distal dendritic compartment of CA1 pyramidal cells. Neocortical CR cells, in contrast, vanish almost completely long before maturity (Del Río et al., 1995; Anstötz et al., 2014), rendering them an unlikely source of adult Reelin. The loss of neocortical CR cells could here be compensated by cortical interneurons, which begin to express Reelin with maturation (Alcántara et al., 1998; Pesold et al., 1998; Pohlkamp et al., 2014).

Because E2 promotes the expression of Reelin (Bender et al., 2010), we hypothesized that E2 mediates its effects on HCN1 distribution via Reelin. However, our data do not support this hypothesis, because inhibiting Reelin signaling using GST-RAP in concentrations that lower Dab1 phosphorylation to 72% of controls (Fig. 2*A*; see also Groc et al., 2007) did not markedly reduce the distal dendritic HCN1 enrichment that was observed after E2 treatment. Moreover, GST-RAP alone did not have an effect, nor did Reelin-blocking CR-50 antibodies (Ogawa et al., 1995) alter HCN1 distribution in our cultures. As these findings are discrepant with the findings of Kupferman and colleagues, and because these discrepancies could have been caused by different experimental conditions *in vitro*, we additionally examined an experimental mouse model in which Reelin was absent *in vivo* (Lane-Donovan et al., 2015). In these mice, deletion of the *reelin* gene was induced by tamoxifen at ∼2 months of age, i.e., after hippocampal maturation is completed, resulting in absence of Reelin during adulthood in a hippocampus with an otherwise normal cytoarchitecture (Fig. 3*C*). Notably, an altered distribution of HCN1 or TRIP8b was not detectable in CA1 of the Reelin-deficient mice (Fig. 4). Taken together, these findings argue against an essential role of Reelin for the establishment and the maintenance of the somatodendritic HCN1 gradient in CA1.

What could be the reasons for the discrepancies between our study and the data by Kupferman et al. (2014)? These authors have based their conclusion mainly on two observations. (1) After viral knockdown of Dab1 in mice, HCN1 expression was selectively diminished and I_h_ reduced in CA1 slm and in the distal dendrites of neocortical layer 5 pyramidal neurons. (2) The HCN1 gradient in CA1 of organotypic hippocampal cultures was abolished after treatment with GST-RAP. In our opinion, both results could be interpreted differently: Dab1, although generally considered a mediator of Reelin signaling, has also alternative functions, as its phosphorylation (i.e., activation) can be induced independent of Reelin, either through LRPs via alternative ligands (Blake et al., 2008; Bock and May, 2016) or through routes that do not require the LRPs, e.g., via amyloid precursor protein (APP; Trommsdorff et al., 1998; Homayouni et al., 1999; Howell et al., 1999; Young-Pearse et al., 2007; Peterziel et al., 2011). Thus, while Kupferman et al. (2014) may be correct in claiming an involvement of Dab1 in the regulation of HCN1 distribution, their conclusion that this is Reelin mediated is not obligatory. Similarly, RAP is a universal inhibitor of low-density lipoprotein receptors that, besides blocking Reelin signaling (Gong et al., 2007; Groc et al., 2007), also binds to other members of the LRP family (Herz et al., 1991; Bu and Schwartz, 1998; Hiesberger et al., 1999), including, e.g., Lrp2/megalin, which mediates the endocytic uptake of retinoids and steroids (Willnow et al., 1999) and is expressed in hippocampus (Alvira-Botero et al., 2010). RAP effects, unrelated to Reelin, may therefore have contributed to the results observed by Kupferman et al. (2014), particularly because high RAP concentrations were applied. Clearly, differences regarding the experimental conditions may have contributed to the observed discrepancies *in vitro*, but these cannot account for the observed absence of an effect of *reelin* deletion *in vivo*.

Whereas Reelin was not required for HCN1 distal dendritic enrichment in CA1, E2 promoted this enrichment via a mechanism that employs GPER1. Estrogens have previously been shown to modulate I_h_ in kisspeptin-expressing neurons of the hypothalamus (Piet et al., 2013; Zhang et al., 2013) and in Ah-type visceral ganglion neurons (Qiao et al., 2013). In both experimental settings, effects were mainly attributed to E2-induced changes of HCN1 expression levels (Qiao et al., 2013; Zhang et al., 2013), which in the visceral ganglia also involved GPER1 (He et al., 2015). Thus, whereas GPER1-mediated regulation of HCN1 channel function has been shown before, the novelty of our findings lies in the fact that in CA1 this regulation influences the subcellular distribution of the channels. The underlying mechanisms require further elucidation but could include E2-induced modulation of HCN1 binding to the cytoskeleton, which tightly controls HCN1 trafficking and membrane integration via microtubule- and actin-associated mechanisms (Noam et al., 2010). In this scenario, GPER1 could be connected to the cytoskeleton through a variety of intracellular signaling mechanisms, as it activates, e.g., the mitogen-activated-protein kinase cascade (MAPK; Filardo et al., 2000; Denley et al., 2018) and phosphoinositide-3-kinase (PI3K; Ruiz-Palmero et al., 2013), which also takes part in Reelin signaling (Jossin and Goffinet, 2007; Chai et al., 2009). E2 may further influence the HCN1 channels’ association with TRIP8b, which controls HCN1 compartmentalization in a TRIP8b isoform–dependent manner (Lewis et al., 2011; Piskorowski et al., 2011; Wilkars et al., 2012) and is sensitive to environmental changes (Lyman et al., 2017; Frigerio et al., 2018).

In summary, our findings suggest that the subcellular distribution of HCN1 channels in CA1 is influenced by estradiol but does not require Reelin, as previously proposed (Kupferman et al., 2014). Why, then, were there no differences evident in female mice of different estrous cycle stages (proestrus vs. diestrus; Fig. 6)? As already pointed out, it must be considered that hippocampal neurons are themselves capable of synthesizing E2, because they are endowed with all enzymes required for steroid biosynthesis, including aromatase, which converts testosterone to E2 (Prange-Kiel et al., 2003; Hojo et al., 2004; Kretz et al., 2004; Fester et al., 2009). In fact, in hippocampal tissue, E2 concentrations are about six-fold higher than those in the blood plasma (Hojo et al., 2004), suggesting that the endogenously synthesized E2 plays an important role in hippocampal information processing. Hippocampal aromatase, however, is under the control of the hypothalamic-pituitary axis, and its activity and expression adjust to the changing E2 plasma levels during the estrous cycle (Prange-Kiel et al., 2008; Kato et al., 2013). The HCN1 distribution in CA1 does therefore not necessarily reflect the fluctuation of the peripheral hormone levels, because—in both males and females—hippocampal aromatase, which integrates signals from the neuroendocrine (hormonal) and neuronal (activity-dependent) environment (Balthazart and Ball, 2006; Charlier et al., 2015; Fester et al., 2016; Hojo and Kawato, 2018), could balance the effects of the peripheral hormones and act as a local mediator of neuroendocrine status and neuronal activity on hippocampal information processing, e.g., by fine-tuning HCN1 channel distribution in CA1.

## Synthesis

Reviewing Editor: Douglas Bayliss, University of Virginia School of Medicine Decisions are customarily a result of the Reviewing Editor and the peer reviewers coming together and discussing their recommendations until a consensus is reached. When revisions are invited, a fact-based synthesis statement explaining their decision and outlining what is needed to prepare a revision will be listed below. The following reviewer(s) agreed to reveal their identity: Tallie Z. Baram.

Synthesis

This manuscript was seen by two reviewers, both of whom were quite positive in their assessment of the work suggesting that estrogen affects HCN1 distal dendritic distribution in CA1 cells, and that published effects of Reelin on that distribution deserve additional scrutiny. However, you will also see from their comments that a number of changes are recommended, particularly in terms of additional or alternative analysis of the data, and further clarification of methods and interpretation.

Notably, please attend to: 1) concerns about antibody staining, providing some negative control data and further description of the characterization/validation of the antibodies employed (as performed in your lab or extant in the literature). Also, quantifying fluorescence intensity is difficult, and conclusions based on those data should be tempered accordingly. 2) A more convincing example to show effects of GST-RAP on pDab:Dab levels would be helpful, since this positive control supports the negative result of GST-RAP on HCN1 distributions (and the conclusion that Reelin is not required for HCN1 distribution).

Please find below the full reviews, that detail the various specific suggestions for improvement.

Reviewer #1

Advances the Field

There are two major advances:

1. Estradiol regulates the unique dendritic distribution of HCN1

2. Prior reports about the role of reelin may not be as solid as thought.

Statistics

I asked the authors to comment on a couple of their methods

Comments to the Authors

The authors address the regulation of the remarkable distribution of the ion channel HCN1 in apical dendrites of hippocampal CA1. Specifically, they address the well described distal dendritic enrichment of HCN1 channels in hippocampal CA1 and demonstrate that this distribution is promoted by estrogen, but does not require Reelin. The premise of the paper is a prominent prior report that HCN1's preferential location in distal dendrites was controlled by the extracellular matrix protein Reelin. The authors fail to replicate these studies in a series of elegant and well-controlled studies in vivo and in vitro. By contrast, they demonstrate a novel role of estradiol in this regulation.

The work is extremely well designed and described, and the experimental results are convincing. A few points deserve the authors' attention:

Results:

1. The authors claim that E2 did not increase HCN1 expression. This statement is not fully supported. They aim to quantify fluorescent intensity, which is a very difficult thing to do. They also conducted western blots - but this was done on whole cultures, and HCN1 is expressed widely in interneurons and other dendritic and axonal compartments within the entorhinal- hippocampal cultures. Therefore, the statement needs to be significantly toned down.

2. During development, an interesting presynaptic distribution of HCN1 in the middle molecular layer of the dentate gyrus has been reported by some of the authorities cited by the authors. Did E2 influence this enrichment? Did reelin? Whereas this paper is focused on CA1, there is an excellent opportunity to enrich our understanding of trafficking and compartmentalization of HCN1 channels. The authors should have the relevant information on hand (indeed enrichment in medial entorhinal cortex is apparent in figure 1).

3. Is there a difference in the distribution of HCN1 between males and females (regardless of cycle)? This would be interesting, even if negative, and might be added to figure 5. Unfortunately, the quality of figure 5B, aiming to show aromatase distribution is insufficient to permit its inclusion.

Methods:

1. It might be helpful to look at ANOVAs when distance from the pyramidal cell layers and a treatment are both factors. ANOVA is more conservative than the methods used by the authors, and an interaction would be quite helpful.

2. Was the same guinea-pig antiserum used for both HCN1 Western and IHC? The same concentration?

3. Minor note: in the organotypic cultures, do the authors use ascorbic acid to enhance culture health?

Reviewer #2

Advances the Field

Demonstrating that 17β estradiol (E2) can modulate trafficking of HCN channels in CA1 pyramidal cells is important new information as both E2 and HCN channels are critical modulators of CA1 pyramidal cell function and hippocampus dependent learning and memory.

Comments to the Authors

This is an interesting study that advances our understanding of the mechanisms stimulated by 17β estradiol that ultimately will modulate CA1 pyramidal cell function. The authors use in vitro entorhinal cortex-hippocampal slice cultures to show the E2 increases the density of HCN channels in the most distal regions of the CA1 dendritic tree. They use a combination of strategies, including Reelin KO mice to show the effect of E2 is independent of Reelin. Using estrogen receptor pharmacological approaches, the authors conclude that E2 is working via GEPR1 to stimulate HCN trafficking to distal dendrites. Finally, the authors interpret data from ovary intact mice at diestrus and proestrus and immunohistochemical localization of aromatase to indicate that locally synthesized E2 in hippocampus rather than ovarian E2 is likely responsible for modulating the HCN distribution in distal dendrites. Overall, these studies were nicely done, but there are needed changes that will improve the quality of this study.

1) It would be helpful to see a negative control for the initial immunohistochemical staining experiments in Fig 1. The background looks high.

2) The Western blot data showing that GST-RAP decreases pDab1 is not too convincing. Potentially it is just the blot that was chosen for the figure. It also looks like there is a decrease in total Dab1, although the authors mention there is no effect of GST-RAP on Dab1 expression. It is critical that these data are convincing since the authors are suggesting that Reelin is not involved because GST-RAP does not prevent the E2-induced increase in distal trafficking of HCN. It would add alot if additional immuno images where shown in Fig. 2. demonstrating that E2 increases HCN in distal dendrites in the presence of GST-RAP.

3) Scale bars are missing from some of the immunohistochemisty images (e.g., Fig. 3A). There are also asterisks missing on some of the bar charts (e.g., Fig. 2b).

4) There is no mention as to how the estrus cycle was determined. If this was done using vaginal smears, it would have been nice to see pictures confirming estrous cycle phase. Without that, it is difficult to be convinced that the mice were at different phases of the estrous cycle.

5)The authors are correct in that current understanding is ovarian E2 indirectly regulates hippocampally synthesized E2. Therefore, showing that pharmacologic aromatase inhibition in hippocampus in vivo modulates HCN localization would significantly add to the story.

6) Why are the results from G1 and E2 a bit different? G1 decreases HCN in segments 1 and 2 and increases in 4, while E2 only increases in 5 with no significant decrease in segments 1 and 2.

7) There are a few places where the sentence structure could be better. For example, in the abstract line 36, "Due to lack of estrus cyclicity..." is not accurate. The animals did have cycles according to the measures at diestrus and proestrus (although no evidence was provided). What the authors really mean is that HCN localization was not different at difference phases of the cycle. Also line 47-48 could be rewritten for better clarity. In the methods, lines 145, 147, 152, the name of the university was omitted.

## Author Response

Dear Dr. Bayliss,

Thank you very much for your letter expressing appreciation of our manuscript, and for the reviewers' very positive and helpful comments. In the meantime, we have worked on addressing these comments, and have added new data and corrections, where they were requested or required. These changes are marked "yellow" in the revised manuscript (marked) and are outlined in detailed responses to the reviewers' comments below:

General notes:

- In the revised Manuscript, previous Figure 2 has been split into two figures (Figs. 2 and 3), resulting in an altered figure numbering

- RRIDs have been added

- The age of the Reelin cKO- (and corresponding wildtype) mice has been specified: 7-8 months (before: 6-12 months; page 7)

Responses to Reviewer 1:

We very value the reviewers' appreciation of our manuscript. To his/her critiques:

1) "The authors claim that E2 did not increase HCN1 expression. This statement is not fully supported. They aim to quantify fluorescent intensity, which is very difficult thing to do. They also conducted western blots -- but this was done with whole cultures, and HCN1 is expressed widely in interneurons and other dendritic and axonal compartments within the entorhinal-hippocampal cultures. Therefore, the statement needs to be significantly toned down."

The reviewer is, of course, correct. The chosen methods do not permit exclusion of the possibility of an effect of E2 on HCN1 expression. We have therefore modified the respective statement, suggesting altered trafficking rather than altered expression, without excluding the latter (page 13).

2) "During development, an interesting presynaptic distribution of HCN1 in the middle molecular layer of the dentate gyrus has been reported by some of the authorities cited by the authors. Did E2 influence this enrichment? Did reelin? Whereas this paper is focused on CA1, there is an excellent opportunity to enrich our understanding of trafficking and compartmentalization of HCN1 channels. The authors should have the relevant information on hand (indeed enrichment in medial entorhinal cortex is apparent in figure 1)."

We thank the reviewer for this suggestion. Indeed, being aware of a potential presynaptic HCN1 expression in mML, we avoided this layer, when determining background levels, which were strictly measured in oML. Unfortunately, going back to the sections upon the reviewers' suggestion, we realized that HCN1 immunosignal is too low in mML to permit a meaningful analysis. This could result from incomplete perforant path preservation (+ insufficient time for the fibers to regrow) in the cultures at the time of preparation, to which fluorescence fading over time may have added.

3) "Is there a difference in the distribution of HCN1 between males and females (regardless of cycle)? This would be interesting, even if negative, and might be added to figure 5....."

In order to address this recommendation, we have added an age-equivalent male group to the analysis in (now) Figure 6. Although some data were suggesting lower HCN1 levels in stratum radiatum (and, consequently, relatively higher levels in slm) in the males, if compared to proestrus females, this trend did not prove to be significant in the linear regression analysis. We therefore conclude that there is generally no decisive influence of the peripheral sex hormones, suggesting that compensatory mechanisms -- involving hippocampal aromatase -- are active locally. Clearly, this question deserves closer examination, which we will undertake in the future.

4) ...Unfortunately, the quality of figure 5B, aiming to show aromatase distribution is insufficient to permit inclusion.

Please note, that this figure (now Figure 6D) is a confocal micrograph, which in our opinion - in the case of aromatase - better illustrates the difference between the strata than the fluorescence microscopy that was used for the other analyses .

5) "It might be helpful to look at ANOVAs when distance from the pyramidal cell layers and a treatment are both factor. ANOVA is more conservative than the methods used by the authors , and an interaction would be quite helpful"

The use of two-way-ANOVA is problematic in the context of our data. Firstly, it is a general linear model (GLM) and its basic principle would not address all of our questions, because the two-way-ANOVA pools all data points for each distance (treatment independent) and for each treatment (distance independent), and thus a loss of information may occur when applied on our data. We therefore opted for a "comparison of the slopes", which linearly compares regression lines of each gradient and can be seen as an alternative for interaction effects, since distance (compartment) and treatment are both taken into account. Secondly, two-way ANOVA requires normal distribution of the data points and homogeneity of error variances, both of which were often not given in our data samples. Still, we have applied two-way-ANOVA to the most central data of our study. The results, shown below, are in good agreement with the statistical results in our paper. We have not yet included them into the manuscript, though, because they, in our opinion, although generally confirmative, do not provide critical additional information:

E2 vs Ctl (Fig. 1F): There was a significant interaction effect between distance (compartments) and treatment: (F(4, 560) = 9.833, p &lt; .001).

GST vs E2+GST vs E2+GST-RAP (Fig. 2C): There was a significant interaction effect between distance (compartments) and treatment: (F(8, 240) = 2.468, p &lt; .05).

G1 vs Ctl (Fig. 5E): There was a significant interaction effect between distance (compartments) and treatment: (F(4, 200) = 5.239, p &lt; .001).

E2 vs Ctl vs E2+G36 (Fig. 5G): There was a significant interaction effect between distance and treatment (F(8, 195) = 3.217, p &lt; .01).

6) "Was the same guinea-pig antiserum used for both HCN1 Western and IHC? The same concentration?

We used different antibodies for IHC and Western: for IHC, rabbit-anti-HCN1 from Merck Millipore (AB 5884) was used (1:500); for Western, the cheaper guinea pig-anti-HCN1 from Santa Cruz (sc-19706) was used (1:500). Unfortunately, this antibody is not commercially available anymore. However, when tested for suitability before use, it revealed the identical staining patterns, both in IHC and WB, as the Merck Millipore AB. A sentence clarifying this has been added to the "Methods" section (page 8).

7) Minor note: in the organotypic cultures, do the authors use ascorbic acid to enhance culture health?

We did not include ascorbic acid in our culture medium, but did not observe any health problems with the cultures.

Responses to Reviewer 2:

Again, we would first like to thank the reviewer for his/her appreciation of the manuscript and the helpful comments.

1) "It would be helpful to see a negative control for the initial immunohistochemical staining experiments in Fig. 1. The background looks high."

Negative controls for both the in vivo and in vitro HCN1-IHC have been included in Figure 1.

2) "The Western Blot data showing that GST-RAP decreases pDab1 is not too convincing. Potentially, it is just the blot that was chosen for the figure. It also looks like there is a decrease in total Dab1, although the authors mention there is no effect of GST-RAP on Dab1 expression. It is critical that these data are convincing since the authors are suggesting that reelin is not involved because GST-RAP does not prevent the E2-induced increase in distal trafficking of HCN.

In the revised Figure 2, we have included another Blot, which is, however, also not perfect (Dab1 levels do not match, but note that for quantification they were normalized to GAPDH), because it was in fact difficult to have all components - pDab1, Dab1, GAPDH - matching within an analyzed pair. In order to underline that - in the sum - Dab1 levels were not different from those in the controls, we have now included the quantification of Dab1 and pDab1 graphically into the figure (2B). We also would like to emphasize that for us the data from this particular experiment were important, but not crucial, for our conclusion, since even with GST-RAP effects lacking in the experimental setting of Fig. 2, too many questions remained (e. g.: was the concentration of GST-RAP sufficient? how critical is treatment duration?) to conclude at this point that Reelin is not involved. But these results prompted us to scrutinize the original findings from Kupferman and colleagues, as shown in (now) Figs. 3 and 4. By re-wording this paragraph, we hope to have made this aspect clearer (pages 13/14)

3) It would add a lot if additional immune images were shown in Fig. 2, demonstrating that E2 increases HCN in distal dendrites in the presence of GST-RAP."

We have added photographs showing HCN1 immunostaining in a representative culture "triple" to figure 2 (Figs. 2E-G). While it is difficult to clearly appreciate ENHANCED concentration of HCN1 at the hippocampal fissure in slm, REDUCED immunostaining in stratum radiatum, indicating stronger relative HCN1 enrichment in slm, should be visible (and is pointed out) in the E2-treated slices.

4) "Scale bars are missing from some of the immunohistochemistry images (e. g. Fig. 3A). There are also asterisks missing on some of the bar charts (e. g. Fig. 2b)".

Missing scale bars and asterisks have been added.

5) "There is no mention as to how the estrus cycle was determined. If this was done using vaginal smears, it would have been nice to see pictures confirming estrous cycle phase. Without that, it is difficult to be convinced that the mice were at different phases of the estrous cycle."

The cycle stage was determined by visual inspection of the vaginal smears, according to "Hoglund (1972)" and "Byers et al. (2012)". This has been clarified in the "Methods" (page 6). We have further included representative examples of vaginal smear cytology for proestrus and diestrus in (now) Figure 6 (C).

6) "The authors are correct in that current understanding is ovarian E2 indirectly regulates hippocampally synthesized E2. Therefore, showing that pharmacological aromatase inhibition in hippocampus modulates HCN localization would significantly add to the story."

This is an excellent suggestion, which we will be glad to pursue. But because such experiments are time-consuming (requiring animal experimentation permission etc.), we will not be able to present data within a reasonable time frame and would prefer to include them into a separate study (which is already under way).

7) "Why are the results from G1 and E2 a bit different? G1 decreases HCN in segments 1 and 2 and increases in 4, while E2 only increases in 5 with no significant decrease in segments 1 and 2?"

Differences within INDIVIDUAL segments were often small and did not reach the significance level (e. g., for segment 5 in the G1- experiment, for which p=0.051). We therefore regard the linear regression analysis ("slopes") actually the more meaningful statistical analysis, because it sums up ALL differences within the segments, thus highlighting the tendency. We still regarded it important to also show the bar charts, which indicate details of the distribution and illustrate our methodological approach.

8) "There are a few places where the sentence structure could be better. For example, in the abstract line 36, "Due to lack of estrous cyclicity...." Is not accurate. The animals did have cycles according to the measures at diestrus and proestrus (athough no evidence was provided). What the authors really mean is that HCN localization was not different at different phases of the cycle. Also line 47-48 could be rewritten for better clarity. In the methods, lines 145, 147, 152, the names of the university was omitted."

Thanks for the reviewers' careful reading. These parts indeed sounded awkward and have been re-worded in the revised manuscript. The omission of the universities' name was, however, on purpose to preserve the authors' anonymity.
